# *You just want a break from the hatred of failure*: the lived experience of being a student physiotherapist perfectionist and considerations for educators

**DOI:** 10.1007/s10459-023-10287-y

**Published:** 2023-10-12

**Authors:** Jane McKay, Kim Williams, Jennie Stewart

**Affiliations:** 1 School of Health and Life Sciences Learning Development Centre, Glasgow Caledonian University, Glasgow, Scotland; 2 School of Health and Life Sciences, Glasgow Caledonian University, Learning Development Centre, Glasgow, Scotland; 3 The Open University, Scotland, UK

**Keywords:** Perfectionism, Health professions education, Student experience, Phenomenology, Physiotherapy students

## Abstract

Perfectionism is a personality orientation associated with mental health and adjustment problems. Recent evidence demonstrates that perfectionism is widespread among students and on the rise, with recent generations of students placing increasingly more importance on perfection. Whilst the extant literature is vast, it tends to focus on psychopathology and identification of perfectionism correlates rather than the experience of student perfectionism. Furthermore, the education literature is scant and there is a need to understand the deeper processes and nuances of perfectionism, particularly within health professions education where intense study demands, competition to gain entry to educational programmes, and professional cultures may nurture the problem. This phenomenological study explored the lived experiences of ten physiotherapy students as they wrestled with perfectionism in the various facets of their studies. Semi-structured interviews were undertaken, and participants completed log sheets to document perfectionism-related experiences. Idiographic profiles were composed and interview transcripts were analysed, drawing upon features of both phenomenological and thematic analysis. Perfectionism was found to have toxic consequences for the learning experience. Harmful phenomenological experiences included perpetual and excessive achievement striving, punitive self-criticism and health and wellbeing difficulties. A range of sabotaging learning behaviours such as self-handicapping and feedback avoidance was also illuminated, and cultural and organisational influences perceived to foster perfectionism emerged. Findings are discussed in relation to underlying processes and implications for educators. The prospect of findings transferring to other educational contexts is highlighted.

## Introduction

Perfectionism is defined as the setting of rigid and excessively high standards for performance, coupled with self-doubt and critical self-evaluation (Frost et al., 1990; Hewitt et al., 1991). Although it is often confused with the pursuit of excellence, perfectionism is conceptually distinct and represents an extreme form of striving (Gaudreau et al., 2022). Interest in the construct has been ongoing for decades and the literature includes extensive quantitative works, typically drawn from university student samples. These works identify associations between perfectionism and negative psychological and clinical outcomes (see Smith et al., 2022), indicating that university student perfectionism is a serious issue. Indeed, recent meta analyses demonstrate that perfectionism has increased over time among university students (Curran & Hill, 2019; Smith et al., 2019) and it has been described as a growing epidemic (Flett et al., 2022). Given its evaluative context, higher education may be considered a “breeding ground” for perfectionism (Marshall et al., 2008). Understanding the student perfectionism experience is therefore necessary in order to increase awareness among learning professionals and inform teaching practices.

Despite the burgeoning literature on perfectionism, the vast majority of research has adopted cross-sectional designs and tends to focus on pathology (Dobos et al., 2021). Whilst this research provides important knowledge of the correlates of perfectionism in academic settings, few studies have considered how perfectionism shapes the experience of learning as a university student (Dickinson & Dickinson, 2015). Consequently, more qualitative work is needed to augment current understandings and unearth the deeper processes of the perfectionist lived experience (Farmer et al., 2017; Molnar et al., 2023). This study aims to address this gap with a phenomenological approach which seeks to understand the experience of being a student perfectionist within the context of physiotherapy education. In doing so, it responds to the lack of research on how perfectionism shapes the learning experience.

### Perfectionism as a maladaptive construct

Perfectionism is widely accepted as a complex, multidimensional entity with personal and interpersonal facets and various measures (e.g. Frost et al., 1990; Hewitt et al., 1991; Slaney et al., 2001). For example, Hewitt et al.’s (1991) Multidimensional Perfectionism Scale differentiates self-oriented perfectionism (being driven to attain personal standards of perfection), socially-prescribed perfectionism (being driven to attain the perceived standards of others) and other-oriented perfectionism (demanding perfection from others). Although perfectionism scales measure both conceptually similar and different dimensions of perfectionism (Kawamoto et al., 2023), they have been found to load onto two higher-order dimensions (Stoeber & Otto, 2006; Egan et al., 2022). These are ‘perfectionistic strivings’ (rigid pursuit of high standards), which is often considered adaptive, and ‘perfectionistic concerns’ (self-doubts, self-critical evaluation and concerns about others’ judgements of performance), which is considered maladaptive. Accordingly, perfectionism has been described as a “double-edged sword” (Stoeber, 2014), although the maladaptive edge may be “sharper and cut deeper” (Stoeber et al., 2020, p.7). The question of whether perfectionism has adaptive characteristics has been the subject of much debate (Osenk et al., 2020). Research that has considered perfectionistic strivings separately to other features of perfectionism (e.g. self-criticism and doubt) may conflate it with similar concepts, such as conscientiousness or striving for excellence (Hill, 2021; Gaudreau et al., 2022). Indeed, Gaudreau et al. (2022) recently provided compelling evidence for the conceptual difference between these concepts and called for an end to the debate about the adaptiveness of perfectionism.

Supporting the view of perfectionism as maladaptive, there is consistent evidence linking it to maladaptive outcomes including depression (Smith et al., 2018a ), anxiety (Klibert et al., 2015), rumination and worry (Xie et al., 2019), eating disorders (Dahlenburg et al., 2019), exercise addiction (González-Hernández et al., 2022), suicidal ideation (Limburg et al., 2017; Smith et al., 2018b) and early mortality (Fry & Debats, 2009). Consequently, perfectionism has been described as a transdiagnostic variable (Bieling et al., 2004), or perhaps more accurately, a ‘core vulnerability factor’ (Hewitt, 2020), meaning that it is not uniquely involved in any particular psychological condition, but operates across diagnostic boundaries and maladaptive outcomes (Dalgleish et al., 2020; Hewitt, 2020).

### Perfectionism in academic settings

As many as 66% of students are perfectionists (Merrell et al., 2011) and perfectionism has been found to be extremely elevated in 14% of students (Molnar et al., 2020). In their important contribution to the field, Curran and Hill (2019) provided cross-temporal meta analytic evidence based on over 41,000 university students from the US, Canada and the UK, demonstrating that perfectionism has increased linearly over the last three decades. This, they reasoned, reflects socio-cultural changes relating to a rise in meritocracy and associated increases in anxious, controlling parenting, which likely fosters perfectionism. Although initial perfectionism research was conducted in Canada and the United States, extensive international research now demonstrates that perfectionism is a global issue (Flett & Hewitt, 2020).

Despite the prevalence of perfectionism and its relevance to achievement contexts, the literature from academic settings is not as developed as might be expected (Lin & Muenks, 2022; Rice et al., 2016). Generally, research has adopted correlational methods and whilst the aforementioned conflation of concepts makes it confusing, there is strong evidence for the detrimental impact of perfectionism on a range of outcomes in academia. These include: achievement (Madigan, 2019; Stoeber et al., 2020), engagement (Closson & Boutilier, 2017), adjustment (Rice et al., 2015), test anxiety (Bong et al., 2014; Burcas & Cretu, 2020; Vanstone & Hicks, 2019), cheating (Bong et al., 2014; Krone et al., 2012), burnout (Collin et al., 2020; Garratt-Reed et al., 2018; Osenk et al., 2020), procrastination (Bong et al., 2014; Sirois et al., 2017) and student loneliness (Hibbard & Davies, 2011; Wang et al., 2009).

Although this correlational research is important in advancing understanding of relationships between perfectionism and academic outcomes, it is subject to limitations. Firstly, findings are inconsistent relating to the magnitude and direction of associations identified (Burcas & Cretu, 2020) and the cross-sectional designs prevent cause and effect from being drawn. Secondly, the reductive approach may provide an incomplete or fixed understanding (Farmer et al., 2017; Molnar et al., 2023), sacrificing understanding of the construct at the expense of the people who live with it (Hewitt, 2020). As such, correlational studies cannot provide a holistic portrayal of the multiple ways in which perfectionism might shape the student learning experience, nor can it expose underlying mechanisms that explain relationships between perfectionism and academic outcomes (Vanstone & Hicks, 2019). Consequently, there have been calls for more qualitative research (Egan et al., 2013; Farmer et al., 2017; Molnar et al., 2023).

### Perfectionism and health professions students

Research indicates that health profession students experience high levels of stress and mental health problems (Omigbodun et al., 2006; Bogardus et al., 2022), and perfectionism is emerging as a possible explanatory construct. Perfectionism has been strongly associated with stress among students from professions including medicine (Enns et al., 2001), nursing, dentistry, medicine and pharmacy (Henning et al., 1998), occupational therapy (Wagner & Causey-Upton, 2017) and health sciences (Bogardus et al., 2022). Studies in dentistry (Collin et al., 2020) and occupational therapy (Wagner & Causey-Upton, 2017) have reported maladaptive perfectionism prevalence estimates of 35% and 46% respectively. Possible reasons for these high figures could relate to distinctive features of health profession education such as the scrutiny associated with professional body requirements, the additional demands of placement, and the competitive nature of gaining entry to programmes.

Two studies focusing on physiotherapy doctorate students point towards physiotherapy programmes as fruitful contexts in which to understand the student experience of perfectionism. First, Bogardus et al. (2021) reported levels of depression, anxiety and stress that were higher than age matched peers. Although the study was not set within a theoretical framework of perfectionism, interviewees reported perfectionism-related experiences including fear of failure, a sense of inadequacy and peer comparison. Furthermore, Richardson et al. (2022) reported positive correlations between perfectionism and perceived stress. The authors highlighted the importance of understanding how the cultures of health professions programmes might foster or buffer perfectionistic thinking. These studies were, however, hampered by lack of theoretical underpinning, perfectionism measurement (Bogardus et al., 2022) and low sample size (Richardson et al., 2022).

### Qualitative research on perfectionism and limitations

Despite the potential for qualitative research to address gaps in the literature, qualitative studies are scarce. Those that do exist help triangulate existing theory, but they also identify important nuances of the perfectionism experience. For example, in their meta synthesis of qualitative research on perfectionism, anxiety and depression, Egan et al. (2022) identified evidence illustrating how cognitive and behavioural processes such as dichotomous thinking and procrastination can maintain perfectionism. Similarly, in an expressive writing task, Merrell et al. (2011) found that personal expectations, stress and avoidance themes permeated writings among perfectionists. Using a narrative life story model, Ma and Zi (2015) found that perfectionists described their lives with negative tone, while Farmer et al. (2017) illuminated participants’ loneliness and relationship problems in their analysis of narrative interviews. Supporting the idea that perfectionists are concerned with achievement, self-control and recognition for success, in their mixed methods study Mackinnon et al. (2013) reported that perfectionism was associated with status and victory themes rather than communion. Recently, Molnar et al. (2023) identified themes from adolescents’ interviews that captured less well documented perfectionism experiences including social comparison, need for validation and self-worth, and critical internal dialogue.

Collectively, these studies unearth deeper phenomenological experiences of perfectionism, highlighting negatively defined life experiences, avoidance and a need for approval. In doing so, they capture the whole person and provide an authentic insight into how perfectionism impacts life experiences. Nonetheless, this literature is limited by its scarceness and tendency to include heterogeneous perfectionist samples, which may mask the true intensity of experience among those with elevated perfectionism. Given the limitations of the literature outlined, this study aims to respond to calls for more qualitative work and to address the dearth of research on how highly perfectionistic students experience academic study. In doing so, we hope to make a meaningful contribution to this limited strand of education literature.

## Methodology

We position ourselves in the social constructivist paradigm, acknowledging that social reality is multiple and that knowledge is socially constructed (Smith, 2016). Aligning with these assumptions, we selected a phenomenological methodology that would allow us to penetrate the inner worlds of participants and understand the phenomenon: the experience of being a perfectionist whilst undertaking a professional degree in physiotherapy. The aim was thus not to describe objective reality, but to understand participants’ views of the world, as they related to this phenomenon.

The specific approach we used was hermeneutic phenomenology, which stems from the work of Husserl (1913-83) and more specifically, Heidegger (1962). Phenomenology research is concerned with lived experience, or our ‘pre-reflective’ consciousness of life (Dilthey, 1985). In hermeneutic phenomenology, the goal is to convert this lived experience into textual expression that uncovers meaning in a previously unseen way (Van Manen, 2016). In doing so, participant narratives are interpreted within individual contexts since their realities or ‘lifeworlds’ are shaped by the sociocultural context in which experience occurs (Neubauer et al., 2019). Whilst each individual’s experience is unique, share having lived the phenomenon being studied. The task of the researcher, therefore, is to present their understanding of individual experience and draw shared understandings (Bartholomew et al., 2021).

In traditional descriptive phenomenology, the researcher is required to set aside or ‘bracket’ apriori knowledge and assumptions so that they don’t ‘bleed’ into the research (Husserl, 1913-83; Giorgi, 2009). However, hermeneutic phenomenology recognises that researchers cannot step out of their own lifeworlds and so ‘pure’ bracketing can never be achieved (Merleau-Ponty & Smith, 1962). Instead, hermeneutic phenomenologists acknowledge and explore how their subjectivity is part of the research process through reflexivity (Neubauer et al., 2019). As highlighted by Rietmeijer et al. (2021), research rigour can be demonstrated in transparent accounts of methods, which we aim to achieve below.

### Context

The study was conducted within the physiotherapy department of a UK university, which delivers a range of professional programmes. Programmes are delivered for students progressing from school level (undergraduate) and for those with previous experience of higher education (postgraduate). On completion, students can apply for professional registration to practise. Learning is spread across traditional and practice education settings. In the traditional setting a range of teaching methods are used, including face-to-face and online lectures, seminars and professional practice simulations. Assessment tasks include written assessments and clinical skills examinations, which take place in a clinically simulated classroom, commonly in the presence of peers. Wherever possible, practice education occurs within the National Health Service, a free public healthcare system, where students undertake placements.

Given that the researcher is viewed as an instrument of the research (Lincoln & Guba, 1985), details of researchers’ backgrounds should be provided to allow the reader to facilitate interpretations of the data (Elliot et al., 1999). The researchers were two learning developers (JM and KW) who work across the health and life sciences faculty of the university, and a former lecturer (JS) from the faculty’s physiotherapy department. JM and KW, the data collectors, neither teach discipline content nor assess on the physiotherapy programmes. Their role is to support students in developing academic literacies, a position that we considered would not introduce bias or participants’ willingness to share experience. The research emerged from the researchers’ observations of perfectionism across a range of allied health professions programmes, but particularly within physiotherapy. JM has extensive experience of working with perfectionistic learners, of undertaking qualitative research and has published qualitative work in education and health journals. KW and JS are trained in qualitative research and data analysis, have many years’ experience of working with perfectionistic learners and JS has published qualitative work in a range of health journals. JM led the research and undertook the data analysis alongside KW, while JS acted as a critical friend (Marshall & Rossman, 2006) throughout.

### Reflexivity

As the subject of perfectionism is personal to all authors, we recognise the potential for bias throughout the research. Therefore, we engaged in an ongoing reflexive process which involved JM and KW keeping reflexive diaries and engaging in regular discussion about our potential influence on the research. For example, following pilot interviews, we reflected on shared tendencies to occasionally interject, perhaps as a result of personal connections with participants’ narratives. The enhanced awareness gained from this, and the risks of prematurely redirecting the conversation, helped ensure that it was subsequently avoided. Through reflection and ad hoc conversations triggered during the analytic process, we became aware that we each identified more strongly with different forms of perfectionism and tended to view the data through slightly different lenses. This fostered an awareness that prompted deeper contemplation around the meaning of what participants said, resulting in greater analytical rigour, than might otherwise have occurred. Moreover, our personal experiences cultivated an empathy and genuine curiosity, which can lead to a richer analysis (Smith & Osborn, 2008). These interactions with the data demonstrate how the methodology is well suited to researchers with personal experience of an issue who wish to explore others’ lived experience (Walker et al., 2021).

### Participants and recruitment

After institutional ethics approval was granted by the PSWAHS Research Ethics committee (reference HLS/PSWAHS/16/261), all students enrolled on physiotherapy programmes were emailed about the study (n = 387). Emails defined perfectionism, outlined the purpose of the study, and invited those who identified as perfectionists to participate in an information session. Seventeen respondents attended the information session in which the authors provided more detail on the study and shared personal experiences of perfectionism. Students who wished to participate (n = 10) then provided informed consent.

### Data collection

Students who provided informed consent received log-sheets and were asked to document perfectionism experiences that occurred over the course of the subsequent academic session (12 weeks). Completed log-sheets were returned prior to interviews and enhanced the authenticity of the research by allowing participants to identify what was meaningful to them in interviews (Plunkett et al., 2012).

Face-to-face, individual interviews were chosen since these would facilitate in-depth, first-person accounts of experiences (Smith et al., 2009). A semi-structured interview guide was developed that allowed flexibility in responses. The guide was piloted separately by JM and KW to assess the comprehensibility of questions. It began by exploring participants’ log-sheet entries. This served as a starting point to explore student perfectionism experiences that participants had recorded. Clarification and elaboration probes were used to achieve deeper understandings and explore different facets of perfectionism that emerged. Thereafter, if not already covered, questions explored different aspects of the student perfectionism experience, including classroom learning, assignment preparation and placement. Finally, participants were asked whether there was anything about their course or the profession that influenced perfectionism (e.g. “*What aspects of your course or profession (if any) influence your experience of perfectionism*?”) Interviews were conducted by JM and KW, were audio recorded and lasted for 60 to 90 min.

### Data analysis

There is no prescribed method for phenomenological research and as Van Manen (2007) cautions, devising a methodology that is too structured can stifle the research. Our analysis therefore did not follow a prescribed procedure, but drew upon key features of phenomenological (Van Manen, 2007) and thematic (Braun & Clark, 2006) analysis. As a starting point for immersing themselves in the data, JM and KW transcribed interviews verbatim and noted verbal and nonverbal aspects. The immersion process continued with both researchers reading transcripts until they were intimately familiar with them. Salient features were recorded and summarised to construct idiographic profiles for each participant. These served two purposes: firstly, they provided an overall ‘schema’ for each participant, which retained an idiographic focus and important contextual insights, and secondly, they were sent to participants as a credibility check to ensure that they adequately represented their lifeworlds, as described during the interview (Lincoln & Guba, 1985).

In the next stage, transcripts were inductively coded. This involved moving beyond what was explicitly said and to interpretation, as deeper insights were sought. Here, we straddled objectivity and subjectivity by being critically aware of our own positions and striving to be as open to new understandings as possible (Finlay, 2013), while using experience and theory to inform interpretations (Neubauer et al., 2019). In doing so we developed a robust system for coding, consistent with thematic analysis. This involved JM and KW independently identifying so-called ‘meaning units’, extracts of text which reflect a distinct meaning (Finlay, 2014), for each transcript. Meaning units formed subordinate themes which, based on commonalities, were then clustered together to form progressively higher-ordinate themes. Throughout this process, the first two authors met regularly to agree on coding and the emerging hierarchical framework. When complete, the third author independently reviewed the entire analysis. Suggested changes were considered and discussed until agreement was reached.

### Research quality and methodological rigour

In addition to Elliot et al. (1999) and Lincoln and Guba’s (1985) ideas for enhancing quality in qualitative research, we drew upon Yardley’s (2000) guidelines which have been endorsed for interpretative phenomenological research (Smith et al., 2009). Beyond the development of idiographic profiles and ongoing reflexivity, ‘sensitivity to context’ involved understanding setting, sociocultural perspectives and how these could influence what participants said. For example, we were mindful of our role as staff and the potential for power imbalance. We addressed this by identifying as fellow perfectionists and communicating our own vulnerabilities in the information session. This personal sharing, we believe, fostered a common ground which facilitated rapport building and empathy, both of which contribute to obtaining valid data (Lincoln & Guba, 1985; Yardley, 2000). ‘Commitment and rigour’ were addressed in numerous ways, including: using log-sheets to give participants more influence over what was discussed, credibility checking, conducting pilot interviews, and using consensual validation procedures. We hope that our careful drafting and redrafting and inclusion of illustrative quotes will allow readers to appraise the fit between the data and our interpretations, and that they will judge the ‘transparency and coherence’ of the research positively. Finally, by conducting the research thoughtfully and meticulously we aim to develop how the lived experience of perfectionism in academic settings is understood, offer insights which are of both practical and theoretical utility and thereby meet the criteria for ‘impact and importance.’

### Findings

Ten students (1 male, 9 female; mean age 25.1 years; 5 undergraduates, 5 postgraduates) participated in the study, aligning with various authors’ recommendations for sample size in phenomenological research (Giorgi, 2008; Morse, 1994; Cresswell, 1998) and reflecting our agreement that too much data risks ‘suffocating’ the voices of participants (Bartholomew et al., 2021). All participants agreed with the content of their idiographic profiles as part of the credibility check and no changes were made. We begin by presenting a brief composite summary of idiographic profiles to reflect the context from which the themes emerged.

### Composite summary

Participants shared their experiences with depth and sincerity and were exceptionally articulate, with strong insights into how perfectionism shaped their lifeworlds. They were high achievers, viewed themselves as being highly perfectionistic, and described themselves as “all or nothing” people with a need to feel in control. Although perfectionism was experienced in the physical domain, where striving towards perfect body image and physical health was evident, it was particularly entrenched in one’s academic life and contaminated the student experience. Here perfectionism was energised by a fear of failure rather than the potential for satisfaction following success, and some attributed this to early experiences of harsh educational or home environments. A few participants identified positive aspects of their perfectionism, such as “the drive to do things and do them well”; however, this came at a cost and on the whole, perfectionism was viewed as decidedly negative and disabling. It is not surprising, then, that the lived experience of being a perfectionist student emerged as a rather self-destructive phenomenon, as is reflected in the themes that follow.

Three broad theme clusters and nine higher–order themes were identified (Table 1). Below we provide a synthesis of the sub-ordinate themes from which these overarching themes emerged. Participant quotations (with pseudonyms) are included to allow participant voices to be heard and to resonate with the reader (Elliot et al., 1999). We acknowledge that this is just one way of representing the findings and that others are possible.

**Table 1:** Broad theme clusters and higher order themes that emerged in the analysis

Academic strivings and the perfectionistic inner world	Being in the learning environment and pressures from the outer world	The toll on health and well-being
• The perpetual strive for perfection	• The need to excel for others	• The toll on mental health
• Academic self as ‘not good enough’	• Presence of others and the proliferation of social evaluation concerns	• The toll on physical health
• Inner voice and the sabotage of student success	• Organisational and cultural influences	
• Dwelling on mistakes and the trauma of feedback		

### Academic strivings and the perfectionistic inner world

This overarching theme captured how thoughts and feelings that had a distinct inward focus shaped the lifeworld and defined how participants were within it.

#### The perpetual strive for perfection

The strive for perfection manifested in an ever-present internal pressure to meet perfectionistic academic standards. Imani, for example, recalled that the only time she had “ever been really happy” was when she received 100% in her placement. Similarly, Rachel was “terrified” of not achieving a first-class degree classification. She said, “*I want it and I make myself sick feeling like I have to get it*.” In their endeavours, participants engaged in over preparation and meticulous study. There was a belief that one had to “read every single word” of extensive reading lists and a tendency to “get caught up in the detail.” Not only was learning approached with a forensic level of detail, there was an incessant and burdensome “need to understand *why.”* This approach to learning was painfully time consuming:

*“I noticed that when we’re doing tests in practical classes I need to understand things in great detail. I can’t just be like, ‘Oh do it this way”; I need to understand why in real detail and I’m much slower than everyone else”* [Natalie].

Perfectionism was especially rampant in the experience of writing. There was no sense of gratification or flow as the constant search for the “right” words and need to “go over and over” work meant that one could “never just write.” Single sentences were typically agonised over and compulsively rewritten in the bid for perfection. This seemingly endless process of iteration was crippling and often culminated in inertia:

*“I will literally spend an hour on just one sentence and if I don’t like it either I’ll stop doing what I’m doing until I get it, or I’ll just stare at it until I can figure out how I can write it”* [Polly].

#### Academic self as “not good enough”

Academic self as ‘not good enough’ was a pervading “feeling inside” of self-doubt and criticism despite objective success. Self-doubts saturated all areas of academic endeavour, with a general sense that one was not doing things correctly and a need for “constant reassurance.” Polly explained:

*“In the student trainer programme it was like me always confirming over and over what the physio wanted me to do to make sure I did it right; I’m always doubting myself until I confirm it with somebody.”*

Feelings of inadequacy and doubt were further reflected in imposterism (e.g. “*In my head I’m like, ‘I don’t deserve to be on this course*’” [Kate]), while the rigidity with which performance was assessed was evident in dichotomous thinking. For instance, Rachel believed that not achieving a first-class degree classification would simply mean failure, even if she secured a job:

*“…the biggest goal for me is to get the first and if I don’t get it, it won’t mean that I can’t get a job, it’ll just mean for me that I wasn’t good enough.”*

Co-existing with self-doubt was a self-critical stance. Feelings of inadequacy seemed to precipitate an immersive spiral of negative thoughts and criticism, as Lewis explained:

*“I just start to doubt my knowledge and stuff. I’m like, ‘Oh is that right?’, and then it just spirals out of control. I just start to heavily criticise myself… and it just sort of snowballs like that.”*

In the case of personal standards not being met, a cascade of self-abuse was unleashed. For Kate, this criticism took the form of “*shame, guilt, a bit of self-loathing …and disgust*.”

#### Inner voice and the sabotage of student success

While ‘academic self as not good enough’ concerned the experiences of self-condemning thoughts, ‘inner voice and the sabotage of student success’ captured how such thoughts led to maladaptive learning strategies and disrupted performance.

Self-handicapping, the paradoxical sabotage of one’s academic performance, emerged from participants’ narratives. This included a range of maladaptive strategies, most commonly procrastination. Participants were cognisant of the reasons for their procrastination, explaining that it provided a way of avoiding the tormenting fear of failure:

*“I just think,’ I’m never going to get this done; as soon as I start I’m going to realise how bad I am at it. Maybe I can just put off feeling like that for a little longer’”* [Imani].

Polly described a sense of being paralysed *as“all the knowledge leaves* [her] *body”*, while Kate said:

*“I just tell myself that I need to take a break and then that break lasts far too long because I just can’t find the courage to go back, but it kills me. It’s like, painful.*”

Other self-handicapping strategies included sabotaging one’s performance in evaluative situations For example, Rachel claimed, “…*every time I get towards the end mark I just seem to flump it*.” Similarly, Hannah explained:

*“…in my exam two days ago, I was being asked to palpate a muscle of the rotator cuff and I don’t know why but I went with the wrong movement, yet I’ve done that movement so many times I could do it with my eyes shut. I think it’s the fear of not doing well, so then I almost like cause it before it happens.”*

Classroom learning and the experience of losing ‘flow’ was another significant experience. The ability to completely immerse oneself in learning was not recognised as a personal strength. Instead, the dominant narrative was about the inner panic and catastrophising that proceeded struggling to grasp learning. Rather than appraising such transient confusion as a normal part of learning, attention shifted from the external to internal as one became “so hung up in [their] inner world”:

*“I get absorbed in lectures so I can be in the flow, but what happens when I don’t understand? The flow stops and then it’s negative emotions like, ‘Oh, I’m not good enough, I can’t pass this exam, and it’s causing all these negative emotions and I’m thinking long-term, like, ‘Oh, am I going to be a good physio?* [Natalie]*’.”*

Entangled in the inner voice was fear of failure and a preoccupation with outcomes. The belief that “all that matters is the grade” contributed to the perceived pressure to excel and meant that the process of learning was undervalued. Similarly, fear of failure prevented “thinking clearly” in performance situations. Hannah said:

*“…during placement, the actual worry and anxiety that feeds in and side tracks me from what I’m doing can disable me.”*

For many, this impeding fear of failure destroyed the experience of being a student:

*“…it just makes you look forward to it being over because you can’t enjoy being at uni studying because you just want a break from the hatred of failure”* [Rachel].

#### Dwelling on mistakes and the trauma of feedback

Engaging with academic feedback was a threatening prospect. There was a sense that accessing feedback would let loose the inner voice and subsequent spiral of self-demise. To avoid such trauma, feedback was often ignored. Abbie said:

*“I hate when you get the email to say the marks are available and I just feel sick. I’d rather not know so I just put it off.”*

When feedback *was* accessed, there was a preoccupation with perceived failures, even in the case of high achievement. For Rachel, her thinking turned to, “*What can I dread next*?”, while Imani said:

*“I’ll always focus on the negative, I’m always thinking, ‘I could’ve done that better’, even though I might’ve got an A+.”*

Where expectations were not met, rumination and emotional responses were severe: “*Somehow it goes inside me and the emotions take over”*, said Natalie, while Kate recalled:

*“I remember in second year I wrote this essay and I thought it was one of the best things I’d ever written but I got like 67% and I cried my heart out; you’d have thought someone in my family had died.”*

Even constructive feedback was hard to take and penetrated to the core. Abbie said, “…*it’s just really hurtful. It feels really personal to me rather than just a critique.”*

### **Being in the learning environment and pressures from the outer world**

Whilst the previous overarching theme concerned how inner thoughts defined experience, this theme related to how external influences shaped experience.

#### The need to excel for others

Perfectionistic striving was reinforced by the sense that one must achieve high standards for others, especially teaching staff. Staff were perceived to reinforce high standards by modelling high accomplishment and even representing “idols” for some. Kate, for example, explained:

*“There’s one lecturer who at uni was expected to get 85% or above to pass…I think she gives off quite a high pressure without knowing it and when she teaches she kind of goes overboard in the amount of stuff she covers.”*

For some, performing perfectly was a way of maintaining self-worth and approval. Rachel said:

*“I’ve sort of set up these higher standards because I thought it would help me to show people that I am worthy.”*

Family members and society at large also contributed to perfectionistic standards. This was especially significant for Hannah, who as only the second person from her family to go to university, felt that if she did not excel she would be “completely disappointing everybody.”

#### Presence of others and the proliferation of social evaluation concerns

Being in the learning environment ignited negative social comparisons where one typically judged oneself to be of a lower standard than peers. Accompanying this was a tendency to ‘mind read’ the judgements of others. This presented a particular problem in clinical skills examinations where peers were present. Abbie said:

*“…when my friends are there watching I’m like, ‘Well they’re really good at this’, and I care about their opinion and they’re just going to be thinking, ‘What is she doing?’”*

Similarly, in relation to clinical assessment, Rachel said:

*“I think that the person assessing me is going to think I’m an idiot, or they already do, eh, that they’re going to compare me to other students and think that I’m so much worse than they were.”*

Social evaluation concerns extended beyond assessment contexts and into the learning arena. Hannah recalled her response to a classroom peer review activity:

*“…it was like my best friend in the class but I couldn’t even let him read my work. He was like, ‘Get a grip!’ I had to let him read it and I literally couldn’t read his because I was too busy worrying about what he was thinking.”*

#### Organisational and cultural influences

Participants’ narratives gave insights into how structural aspects of the learning environment shaped the student experience and gave rise to organisational (learning and assessment formats) and cultural (related to the course and profession) themes. The most significant organisational theme was the format of clinical skills examinations and classroom practice, where the presence of a peer patient simulation model, or having to act as one, triggered intense perfectionistic concerns. For Kate, the fear of making a mistake in an assessment in front of “a second-year model who knows everything” made her “mind spiral and get really blurry.” For Hannah, her anxiety centered on body image evaluation. Specifically, when having to remove items of clothing in practice education she became “*so stressed about whether other people were looking over at* [her].” Attending to such concerns while processing assessment tasks or learning content resulted in cognitive overload and, in the case of examinations, underperforming.

Group work tasks, where one had to relinquish control and tolerate others’ lower academic standards, also emerged as significant. Where tasks were assessed, the reliance on others was threatening to performance. Ilona said:

*“I feel like other people can’t live up to my standards and I don’t want my mark to be pulled down by other people’s lack of effort.”*

In practice education, perfectionism was aggravated by the unpredictability of professional interactions with patients. Ambiguity around patient presentation generated discomfort and a sense that one was not in control. For Kate, this was unnerving, as is captured in a moment of enlightenment:

*“I just realised that’s why physiotherapy is so scary for me, because I’m not in control. Oh my God it makes so much sense; because I have to see a patient and I have no idea of what’s wrong with them and so I’m not in control!”*

Cultural influences that fuelled perfectionism included competitiveness and perceptions and pressures associated with the social status of the profession. Competition was articulated as peers being uncollegiate and vying for grades, attention from staff and jobs at the end of the programme. Rachel said:

*“…coming into the programme and seeing that it’s a competitive environment has really fed into things.”*

Physiotherapy was perceived to have an esteemed public image, positioned as the “best profession” within a “definite hierarchy.” Indeed, some participants had chosen their course because they viewed physiotherapy as a “high achieving career.” These perceptions brought additional pressures to live up to societal expectations, including a sense that one must have solutions to all physical problems:

*“… in some countries we’re really respected and we look like gods; we have this pressure from society to live up to that perception, and when people say, ‘Ah you’re a physiotherapist’, and I think, ‘I should know that’, I know logically I can’t know everything but still people expect you to know so I think society plays a huge role”* [Natalie].

### The toll on health and well-being

This theme related to the mental and physical health problems that participants experienced.

#### The toll on mental health

Mental health problems were mentioned frequently and included: difficulties coping with extreme emotions and self-criticism, depression, anxiety, and having well-being contingent on achievement outcomes. Participants spoke of how overwhelming thoughts and emotions eclipsed the ability to think rationally and self-soothe. This typically occurred following perceived failure. Polly said:

*“It just comes back to anxiety and I get really worked up and think about it [not meeting expectations] lots and I just, sorry if I get worked up, I just get really down.”*

For many, mental health problems manifested in anxiety linked to high self-expectations. Here participants described “crumbling” under the pressure to achieve and fear of “not being good enough”, or of inescapable negative emotional responses to failure. Kate shared:

*“Sometimes I just, like, take myself away…. I just think, ‘I can’t deal with this anymore, I’m so sad; I just put so much pressure on myself.”*

Such pressure had prompted some to seek medical advice and three participants had received a medical diagnosis of depression, which, like others, Hannah attributed to being “genuinely so stressed” and putting herself “under so much pressure.”

Participants also explained how their sense of wellbeing and self-worth was contingent on external outcomes, including positive feedback from the social environment and objective success. Rachel summed this up as follows:

*“…there’s no chemicals telling me to be stressed; it’s me saying that if I don’t achieve I’m going to be unsuccessful and to be unsuccessful is one of the worst things I could think of.”*

#### The toll on physical health

Health issues also manifested physically. There was a sense that relaxation could not be afforded, and excessive striving and associated anxiety led to sleep deprivation. Natalie recalled a recent experience of assignment writing:

*“I had to do lots of references and it took me like 6 hours and I was going to bed at 3am, and I know I could have finished at midnight and it would’ve been alright but I couldn’t; it just had to be perfect…I had like 2 hours sleep because in the morning I had to continue.”*

Some participants felt that sleep difficulties were responsible for physical problems, which for Lewis included stomach ulcers: “*I burn myself out with the stress and always get ulcers with the lack of sleep*.”

Other physical consequences included feeling nauseous in evaluative contexts and breathing difficulties, including panic attacks. Imani said, “…*sometimes when I get really anxious it’s like I just can’t fill my lungs enough*.” For Kate, physical struggles included a back problem, which she attributed to perfectionistic fears and waves of “*really bad sciatic nerve pain*.” She said:

*“You just feel like you’re not good enough and your life’s just crumbling around you and then the back pain starts playing up.”*

Finally, and unsurprisingly, exhaustion and burnout were common, as Rachel described:

*“It’s exhausting; it just drains your entire life. I’ve been exhausted for months, I’ve just been so, so tired.”*

## Discussion

The purpose of this study was to gain an in-depth insight into the phenomenon of being a student perfectionist undertaking a professional degree in physiotherapy, a phenomenon which has not been published to date. By penetrating and illuminating the lifeworld, the study has exposed perfectionism as a complex characteristic that goes well beyond striving for excellence and is injurious to both learning and health. This was encapsulated in three broad themes relating to participants’ inner world, their perceptions of the outer world and the toll on health and wellbeing.

The inner world was typified by compulsive striving, pervasive self-criticism and doubt, imposterism, ‘all or nothing’ thinking and ultimately, exhaustion. These findings are consistent with self-oriented perfectionism (Hewitt et al., 1991) and various works linking perfectionism to imposter syndrome (Holden et al., 2021), lower academic self-efficacy (Kurtovic et al., 2019) and persistent self-criticism (Merrell et al., 2011). A new insight to the literature was the finding that perfectionists were driven to study in deep, excessively meticulous ways. The inordinate time and energy they invested reflects the extreme lengths that perfectionists will go to, and further stresses the risks of spending excessive time in goal productive tasks (Wagner & Causery-Upton, 2017). Persistence and the use of deep learning strategies is clearly advantageous to learning, and has been articulated as such in various perfectionism studies (e.g. Chasetareh et al., 2023; Mills & Blankstein, 2000; Vogel et al., 2019). However, this study indicates that the learning process is painfully slow, necessitates huge personal costs, and robs perfectionists of any gratification to be gained. No positive themes relating to perfectionistic striving emerged, supporting the view that this dimension of perfectionism is not adaptive (Gaudreau et al., 2022).

Also related to learning, this study suggests that the disruptive influence of negative self-appraisals on concentration can disturb academic flow and cause a shift to an inward, critical focus. Such focus, triggered in this study by failing to grasp learning content, reflects an internal attributional style, which has been reported elsewhere among perfectionists (Farmer et al., 2017). That is, when faced with perceived failures (e.g. not understanding), perfectionists tend to blame themselves rather than the situation or others (e.g. poor teaching, classroom environment). This denies them the self-protection afforded by an external attributional style, highlighting a further psychological risk and likely hallmark of the perfectionist student experience. These findings also support those of Ljubin-Golub et al. (2018), who reported that extreme perfectionism is negatively associated with academic flow and mediated by negative emotional content. Although this particular manifestation has rarely been reported, it may be common among perfectionistic students due to repetitive negative self-evaluation. This study adds depth to this finding and helps explain why perfectionists may have to expend substantial amounts of effort in order to maintain concentration.

Maladaptive study strategies uncovered included self-handicapping, typically in the form of procrastination. Tasks involving writing were especially problematic. Interestingly, this resonates with work demonstrating that perfectionist academics produce fewer articles (Sherry et al., 2010). In this study, writing generated a crippling fear of failure and procrastination was a strategy adopted to protect against such thinking. An alternative explanation for procrastination is its use for self-handicapping, that is, excusing failure through, for example, claiming poor preparation, illness or lack of time (Marshall et al., 2008). This kind of self-handicapping emerged in this study in relation to evaluative situations such as clinical skills examinations. Here, the negative affect associated with performance evaluation was so unbearable that participants deliberately and paradoxically sabotaged performance, perhaps in an effort to seize control and provide an alibi for anticipated failure. These findings support and elaborate on previous quantitative work linking high levels of perfectionism to procrastination (Sirois et al., 2017) and other self-handicapping strategies (Hill et al., 2011). Although such strategies may provide instant relief from punitive self-dialogue, they have negative consequences for health (e.g. Sirois & Molnar, 2016) and academic achievement (Rice et al., 2016). Accordingly, perfectionists could benefit from tailored procrastination / self-handicapping support. We would argue, as others have (Marshall et al., 2008), that superficial strategies (e.g. removing distractions, setting time limits) are not likely to be effective; rather, deeper approaches that address underlying perfectionistic beliefs are required.

The tendency to avoid engaging with coursework feedback was another maladaptive behaviour. Its purpose was to ensure psychological safety by evading judgements that might confirm self-doubt and imposter notions. Although this is an important finding that has clear implications for one’s academic development, it has seldom been reported. Similarly, literature on academic writing and perfectionism is surprisingly scant yet writing was highly problematic for participants in this study. We suggest that academic writing and accessing feedback present similarly threatening contexts because the perfectionist’s sense of identity and worth is attached to achievement (Egan et al., 2022; Hewitt, 2020). So too are the products that contribute to that achievement (e.g. written coursework) and therefore producing coursework and receiving feedback is likely to pose a very real threat to one’s sense of self. These insights highlight another facet of learning with intervention potential, and have implications for how educators construct feedback and emphasise grades and other outcomes.

Whilst the experience of being a student perfectionist was influenced by intrapersonal perfectionistic features, interpersonal features also shaped the lifeworld, aligning with Hewitt et al.’s (1991) socially prescribed perfectionism. In the classroom, although high standards were not necessarily imposed on participants, the perception that teaching staff modelled perfection was enough to trigger externally-driven perfectionistic striving. It is worth noting that for one participant, external pressures were directly related to her status as a first-generation student. Socially prescribed perfectionism involves pressures from society at large (Flett et al., 2022), and the exclusionary culture of university education, with its middle class, white, male norms (Bale et al., 2020), may be a societal pressure that increases this form of perfectionism among first generation and other underrepresented student groups.

Further interpersonal facets of the perfectionist student experience included negative social comparison and self-presentation concerns. This was especially evident in relation to clinical skills examinations where the presence of others (e.g. assessors, peers) heightened social comparisons and anxiety about others’ judgements. Whilst numerous studies link elevated perfectionism to test anxiety (e.g. Bong et al., 2014; Diaz, 2018; Eum & Rice, 2011), few explain the mechanisms involved (Vanstone & Hicks, 2019). With regard to clinical skills examinations, one explanation relates to perfectionistic self-presentation, where individuals engage in impression management in an attempt to avoid criticism by appearing as perfect to others (Hewitt et al., 2003). Cowie et al. (2018) suggests that because of associations between perfectionistic self-presentation and both social anxiety and doubts about social competence, interpersonal communication (as required in clinical skills examinationss) may present a particularly high-risk situation. This could explain the debilitating anxiety, cognitive overload and consequent underperforming described in this study.

Participants were sensitive to cultural and organisational aspects of the learning environment, which provide further insights into how perfectionism might be inadvertently nurtured. The format of clinical skills examinations was a particular issue and although other programmes might adopt different assessment arrangements, findings highlight the need for thoughtful construction of assessment formats. Whilst it is important that students are assessed in scenarios that simulate real practice, reducing the potential for socially driven perfectionistic concerns might be fruitful through, for example, minimising the presence of others and recruiting simulation models unknown to those being assessed. Given the perfectionist’s need for control (Molnar et al., 2023), learning environments that threaten this are also likely to be problematic. Physiotherapy practice, like many other health professions, is, by its very nature, unpredictable, and places students in scenarios which conflict with perfectionistic needs for certainty. Whilst students are likely to learn to deal with this associated discomfort over time, facilitating self-understanding about control needs may prove helpful, as appeared to be the case for one student in this study.

Another aspect of the profession likely to foster perfectionism, which has been noted elsewhere (Turner, 2001), is its perceived social status. In this study, beliefs about physiotherapy’s occupational prestige reinforced perfectionistic standards, a finding that is likely to apply to other health professions. This provides another example of how social influences can foster perfectionism and may be especially relevant in a society which increasingly values upward social comparison and status (Curran & Hill, 2019). Lastly, competitiveness emerged as a problem for participants and has been reported elsewhere among physiotherapy students (Richardson et al., 2022). Our findings suggest that competition among students contributes to the development of perfectionistic learning environments, which may reflect shared trait competitiveness within student cohorts. Although competitiveness and perfectionism are conceptually distinct (Klein et al., 2020), high levels of perfectionism have been linked to hyper-competitiveness (Garanian et al., 2018; Molnar et al., 2023). If these traits are common among students within particular professions, they may work together to intensify perfectionistic environments.

Finally, findings illuminate the toxic experience of perfectionism on health and wellbeing. Consistent with other qualitative studies (Farmer et al., 2017; Molnar et al., 2023), participants reacted with intense negative emotions to perceived failure, which took its toll on health. Compulsive striving and the accompanying self-doubt and criticism also contributed to health problems, thus emphasising that these perfectionism dimensions are reliable predictors of psychological maladjustment (Burcas & Cretu, 2020). Moreover, the tendency to construct one’s self-worth around academic achievement was clear, and supports clinical perfectionism where self-criticism exacerbates anxiety and depression, which reinforces self-worth-achievement dependency, thereby maintaining a cycle of continual striving (Egan et al., 2022). Unsurprisingly, the resultant exhaustion and burnout reported in other works (e.g. Hill & Curran, 2016), was abundantly evident in this study. These findings resonate with Flett et al.’s (2022) perspective that perfectionistic striving is not rewarding, but a distinct pathway to physical, emotional and cognitive exhaustion.

### Strengths / limitations

As perfectionism research has, to date, been largely quantitative in focus, this qualitative study addresses a significant limitation. Not only does it provide rich insights into the phenomenon, it exposes underlying mechanisms, thereby offering new understandings and elaborating on existing ones. Whilst the incorporation of idiographic profiles and procedures to enhance methodological quality ensured a rigorous method, limitations should be acknowledged. Firstly, although we paid careful attention to reflexivity, it is possible that our pre-existing assumptions introduced bias. Secondly, criticism could be levelled at the generalisability of findings by those with different epistemological perspectives. We suggest that findings may well transfer to other educational contexts given the previously discussed published work and our own practice on perfectionism, across a range of healthcare disciplines. Further research with students from different health professions would help to flesh this out. Moreover, in line with Smith et al’s., (2009) argument for ‘theoretical generalisability’, we have sought to offer insights that are authenticated by the resonance they have with the reader’s professional and experiential knowledge. Findings are thus generalisable to other contexts as the reader judges to be the case.

### Implications and conclusion

This study has illuminated a constellation of risks associated with being a perfectionistic student, which are harmful to both the learning experience and health. As such, the findings have some important implications for learning professionals.

One obvious question relates to how perfectionist students can best be supported. Whilst empirically supported approaches (e.g. CBT) can be found in the clinical literature, offerings are scant within education and subclinical populations. At the individual student level, as perfectionists are unlikely to relinquish their high standards due to perceived benefits (Merrell et al., 2011; Egan et al., 2013), this obvious solution is unlikely to be successful. We maintain that progress can be achieved by increasing students’ self-understanding of maladaptive behaviour patterns and challenging core perfectionistic beliefs. For example, our learning development work suggests that fostering self-understanding of the reasons for procrastination and encouraging views of coursework as efforts invested in singular tasks, rather than a reflection of the whole self, can be empowering. In addition, given that perfectionists are more likely to use avoidant emotion-focused coping which can exacerbate anxiety (Vanstone & Hicks., 2019), support that aims to elicit more adaptive coping patterns could prove fruitful. Our experience suggests that carefully facilitated workshops that promote peer support and co-creation of self-help strategies may be beneficial. Given that perfectionists often feel socially isolated (Hewitt et al., 2017), such support might also help to cultivate a valued sense of community and connectedness.

In the classroom and in practice education, it would be helpful for educators to consider how their learning environments might inadvertently foster perfectionism. Climates that nurture unnecessarily high expectations, social comparison, competitiveness, and criticism following mistakes are problematic. Conversely, praising effort, emphasising processes rather than outcomes, modelling that it’s acceptable to make mistakes, and as Marshall et al. (2008, p. 31) suggests, writing “with a green pen” are likely to forge perfectionism-friendly climates. Further, promoting growth mindset cultures, where failure is normalised and construed as an opportunity for improvement (Dweck, 1999), may help perfectionistic students engage with feedback and facilitate reappraisals following perceived failure. Indeed, growth mindset cultures have been recognised as a way of preventing implicit reinforcement of perfectionistic standards in medical students (Puri et al., 2023). It could be argued that this is especially important among health profession students who, due to practice evaluation, are subject to more assessment than most students. Such efforts, accompanied by providing clear expectations, structure, and co-creation opportunities may increase students’ sense of learner agency and would have potential to benefit not just perfectionists, but all students.

Educators could also consider explicitly addressing the problem of perfectionism with students. This could involve embedding core sessions across academic programmes to raise awareness of the consequences of rigid self-expectations and promotion of adaptive perspectives such as ‘good enough’ principle (Ratnaplan & Batty, 2009) and ‘excellencism’ (Gaudreau et al., 2022). Clearly, this would require ‘buy-in’ from course educators and a coordinated approach to cultivating perfectionism-buffering learning environments. In that connection, induction sessions might provide an opportunity to engage students in the co-creation of classroom values and codes of conduct (e.g. collegiality, compassion, gratitude). These areas provide fruitful avenues for further research, alongside identification of student groups who, for various socio-cultural reasons, may be more vulnerable. With perfectionism on the rise and universities offering fertile grounds for its development, the issues uncovered in this study are likely to deepen and persist. It is therefore more urgent than ever to find ways of mitigating its pedagogical and emotional wounds.

## Data Availability

The data that support the findings of this study are available from the corresponding author upon request.
